# Supplementary material for: The Microphenotron: a novel method for screening plant growth-promoting rhizobacteria
Source: PeerJ. 2022 May 13;10:e13438. doi: 10.7717/peerj.13438 (PMC9109696; doi:10.7717/peerj.13438)
Supplement: Supplemental Information 3 — Tube on the left side is control followed by Pro. fasciculus S-3, B. thuringiensis S-26, M. pluranimalium S-29 and P. stutzeri S-80 under controlled axenic condition. [file peerj-10-13438-s003.pdf]

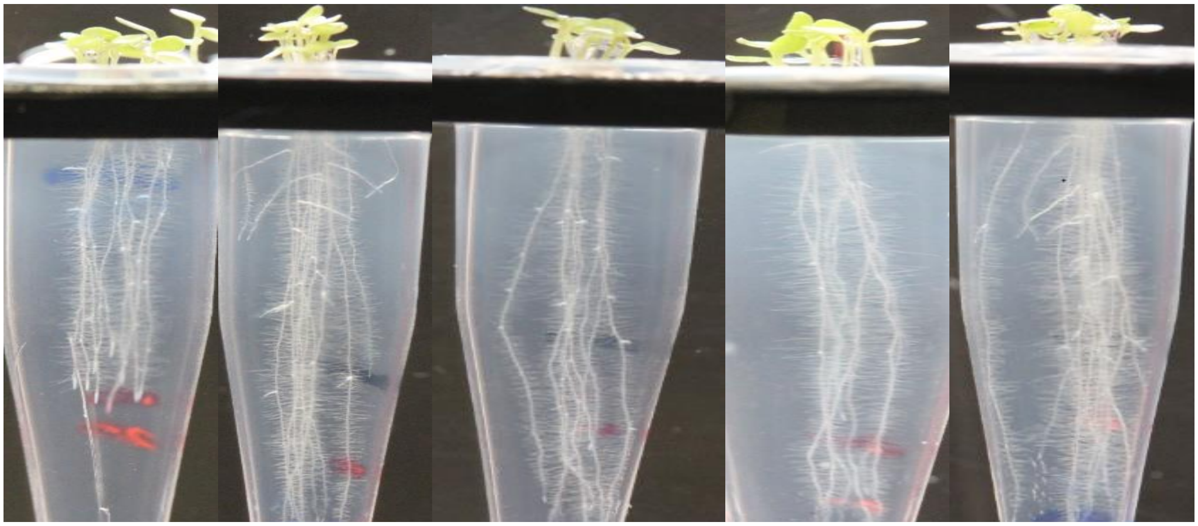

Figure S1. Screening bioassay through novel 96 well PCR plate method. Tube on the left side is control followed by *Pro. fasciculus* S-3, *B. thuringiensis* S-26, *M. pluranimalium* S-29 and *P. stutzeri* S-80 under controlled axenic condition.
