# Supplementary material for: The Microphenotron: a novel method for screening plant growth-promoting rhizobacteria
Source: PeerJ. 2022 May 13;10:e13438. doi: 10.7717/peerj.13438 (PMC9109696; doi:10.7717/peerj.13438)
Supplement: Supplemental Information 4 [file peerj-10-13438-s004.pdf]

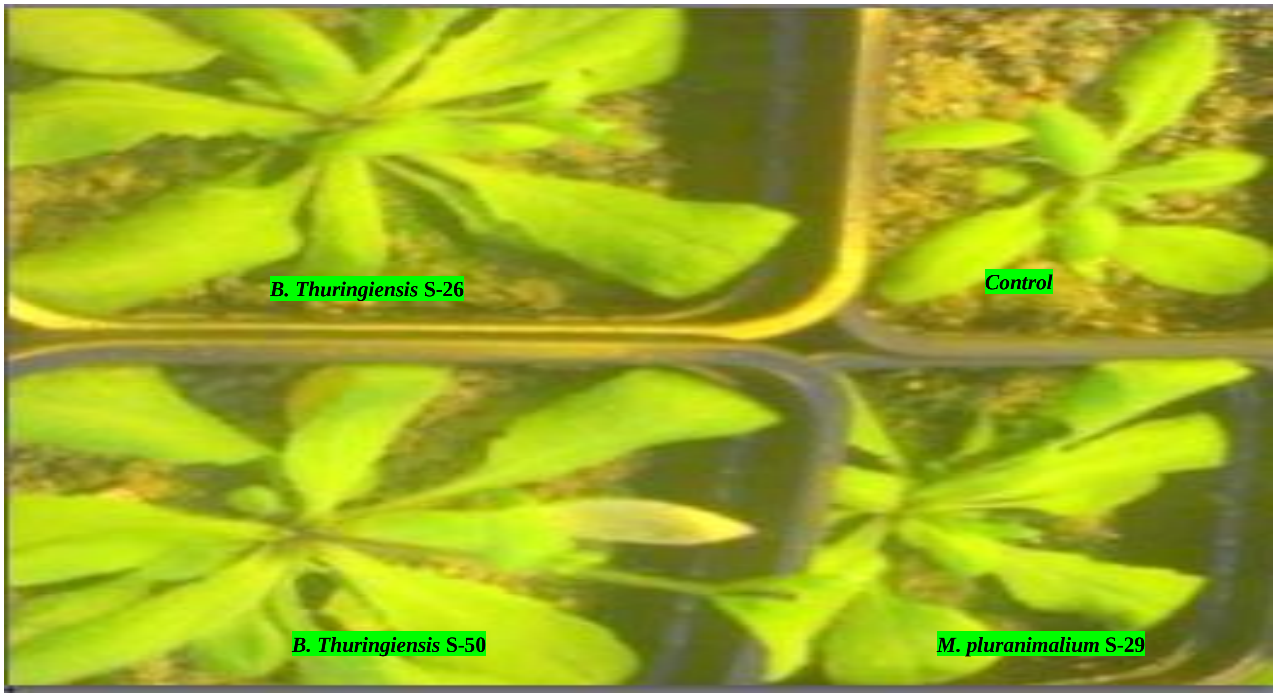

Figure S2. Pot trials with *A. thaliana* wild type (Columbia N6000), inoculated with *B. thuringiensis* S-26, *M. pluranimalium* S-29 and *B. thuringiensis* S-50 in comparison with un-inoculated control
